# Instability of the Pelvic Ring: A Special Clinical Entity?

**DOI:** 10.3390/jcm9061985

**Published:** 2020-06-25

**Authors:** Peter V Giannoudis

**Affiliations:** Academic Department of Trauma & Orthopaedics, Leeds Teaching Hospitals, University of Leeds, Leeds LS1 3EX, UK; pgiannoudi@aol.com; Tel.: +44-113-3922750; Fax: +44-113-3923290

The presence of the pathological movement of pubic symphysis under normal activities characterises a syndrome know as anterior pelvic ring instability [1]. The true incidence of this clinical entity remains unknown. In addition, the aetiology of this syndrome can be multifactorial. Pregnancy related pubic symphysis rupture, disruptions of traumatic nature that were managed non-operatively, hypermobility syndrome, inflammation (pubis osteitis), degenerative conditions secondary to overuse injuries, systemic inflammatory diseases, and prior urological or gynaecological interventions can all lead to anterior pelvic ring instability [2].

The induced painful stimuli are usually localised in the suprapubic region and inner thigh areas, but they can also affect the sacroiliac joints and the buttocks. Such activities as walking on abnormal surfaces, going up and down stairs, side lying, sexual intercourse, running and standing on one limb can aggravate the symptoms.

Diagnosing the syndrome requires a thorough history and a detailed clinical examination. Provocative tests of stressing the pubic symphysis and sacroiliac joints would elicit irritability, as well as deep palpation to the suprapubic area and symphysis. Clinical examination should exclude such pathologies as inguinal/abdominal hernia, trochanteric bursitis, hip impingement and urologic or gynaecological conditions. 

Radiographic investigations would help, not only to confirm the suspected diagnosis, but also to exclude other underlying bone pathologies. Acquisition of supine pelvic views may demonstrate bone pathology such as the non-union of previous sustained pubic rami fractures, chronic osteomyelitis and the presence of metastatic disease. However, being static, instability will be missed. Consequently, dynamic stress views are essential to detect instability. The so-called ‘flamingo views’ (single limb standing views) will demonstrate the degree of movement (described as cranial motion of the weight bearing hemipelvis by the comparative displacement of the pubic body at the pubic symphysis in relation to the opposite side) [3]. The vertical displacement amongst the two pubic bones computed in each single stance radiographic view is added to establish the overall existing displacement. Studies have shown that the normal degree of movement was higher in women—1.5 mm, compared to men—0.5 mm. Other authors have reported that up to 3mm might be a usual finding in women with more than two pregnancies [4].

Computed tomography can delineate bone pathologies related to dysmorphism, osteomyelitis and degenerative conditions of the sacroiliac (SI) joints, whilst MRI can help in the diagnosis of inflammation, oedema, subtle infection, degeneration, bone metastasis and overuse tendon injuries. Interestingly, the dynamic evaluation of the pelvic ring ligaments with MRI has not been investigated as yet.

The management of anterior pelvic ring instability should take into account the underlying pathology, as well as any patient’s specific demands and expectations. Non-operative measures include bed rest, physiotherapy (focusing of pelvic floor strengthening exercises), alternation of activities, pain relief strategies (medication, acupuncture, nerve denervation), pelvic binders, and guided injections of local anaesthetics with corticosteroids. Of note, these have also been used preoperatively to justify surgery. Interestingly, the methods of guidance vary (fluoroscopy and/or CT), and whilst allowing targeted delivery of substances to the pubic symphysis and sacroiliac joints, there is no robust evidence that one substance is superior to the other. 

Failure of the above measures leads to operative intervention, in the form of pelvic fusion. Pubic symphysis fusion is usually an open procedure involving debridement of the joint cartilage, bone grafting and plating fixation of the symphysis [5]. The use of a tricortical bone graft harvested from the iliac crest has been described to reduce the risk of failure [6]. For the SI joint fusion, both open procedures (anteriorly and posteriorly) and minimal invasive techniques have been described. Good results have been reported, although the open approach provides better visualisation of the joint and cartilage debridement prior to the implantation of bone grafting material [7].

Despite extensive clinical interest and the availability of a number of related publications, there is still a lack of consensus regarding the incidence, clinical manifestations, pathophysiology, diagnostics, treatment algorithms and final outcome of surgical reconstruction.

Consequently, both clinicians and scientists are invited to contribute their work in the special issue in the *Journal of Clinical Medicine*, focusing on this important clinical entity, with the ultimate aim to further advance our understanding of the pathophysiology of this condition and the results of surgical treatment.
